# Color overlay of contrast-enhanced endoscopic ultrasound for pancreaticobiliary disease

**DOI:** 10.1055/a-2164-0850

**Published:** 2023-10-06

**Authors:** Haruka Toyonaga, Tsuyoshi Hayashi, Masayo Motoya, Toshifumi Kin, Kuniyuki Takahashi, Akio Katanuma

**Affiliations:** Center for Gastroenterology, Teine Keijinkai Hospital, Hokkaido, Japan

Contrast-enhanced endoscopic ultrasound (CE-EUS) has been considered an important examination for visualization of blood flow and for its contribution to more accurate diagnosis in various conditions of the pancreaticobiliary region 1 2 3 4 5 . However, the conventional black and white mode may limit visual discernibility. A new EUS processor (EU-ME3; Olympus Co., Tokyo, Japan) has been equipped with a novel color overlay mode, which could potentially augment the perception of contrast agents, enhancing the utility of CE-EUS. We present three cases in which the color overlay mode of CE-EUS improved visualization during observation and tissue acquisition ( Video 1 ).

**Video 1** This video presents three cases in which the color overlay mode of contrast-enhanced endoscopic ultrasound improved visualization during observation and tissue acquisition.


Case 1: CE-EUS was performed for a patient with intraductal papillary mucinous neoplasm with nodules ( Fig. 1 ). The color overlay mode offered a far clearer visualization of the contrast-enhanced nodules within the cyst, compared with the conventional mode ( Fig. 2 , Video 1 ).

**Fig. 1:**
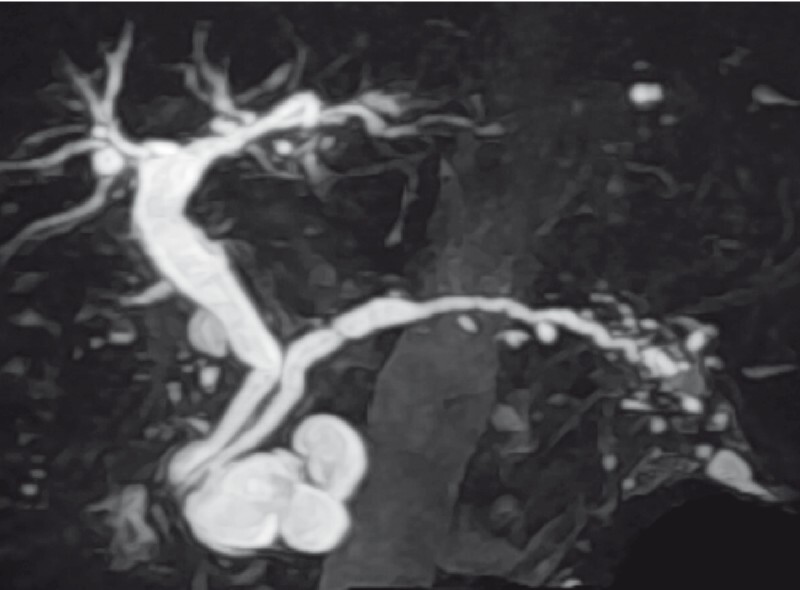
Case 1: Magnetic resonance cholangiopancreatography image of a branch duct intraductal papillary mucinous neoplasm in the pancreatic head.

**Fig. 2:**
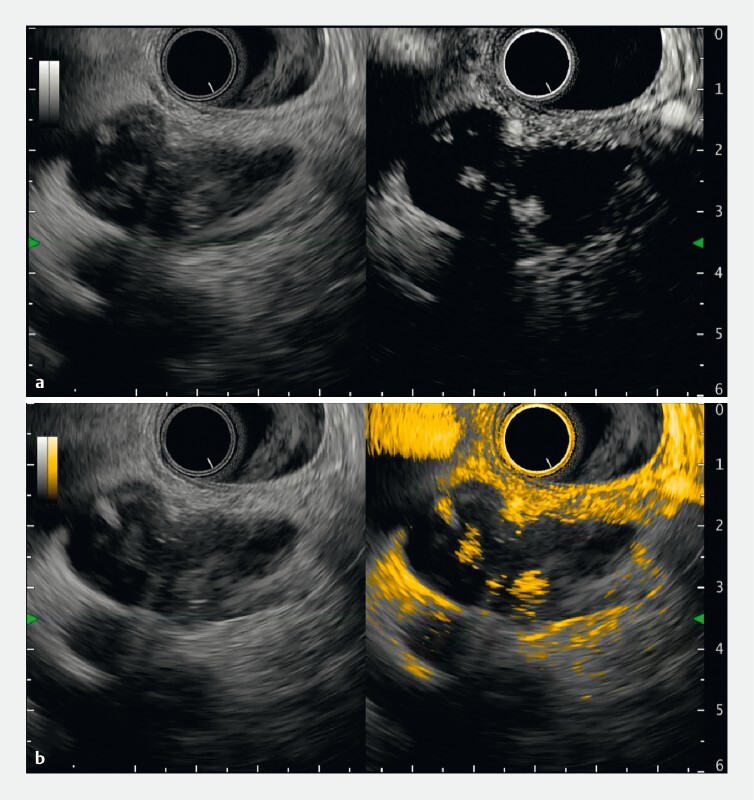
Case 1: Contrast-enhanced endoscopic ultrasound indicated enhanced mural nodules in the branch duct intraductal papillary mucinous neoplasm. **a** Left: B-mode; right: normal contrast-enhanced mode. **b** Left: B-mode; right: color overlay mode.

Case 2: A patient with intrahepatic cholangiocarcinoma whose lesion localization and boundaries were unclear with various imaging modalities underwent CE-EUS for observation. By utilizing color overlay mode, regions devoid of contrast agent within the tumor were better delineated, facilitating lesion localization ( Fig. 3 ).

**Fig. 3:**
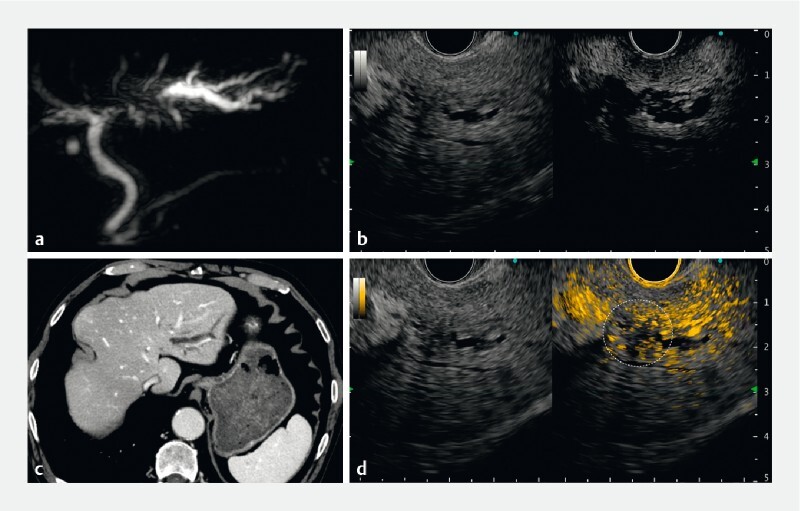
Case 2: **a** Magnetic resonance cholangiopancreatography indicated left hepatic duct obstruction. **b** Contrast-enhanced computed tomography indicated obstruction and upstream dilation of the left hepatic duct; however, no obvious mass could be noted in the obstructed area. **c** Left: B-mode; right: normal contrast-enhanced mode. **d** Left: B-mode; right: color overlay mode. The numerous adjacent vessels and dilated bile ducts made it difficult to recognize the mass lesion in the conventional black and white contrast-enhanced endoscopic ultrasound mode. On switching to color overlay mode, hypovascular areas without orange contrast particles appeared and the lesions causing biliary obstruction could be identified (dashed circle).

Case 3: A patient suspected of having an expansile necrotizing tumor in the head of the pancreas was scheduled for EUS-guided tissue acquisition ( Fig. 4 ). Viable tissue sampling is paramount to enhance diagnostic yield. However, discernibility was challenging on conventional CE-EUS. By applying color overlay mode, contrast particles were clearly identified, leading to efficient sampling ( Fig. 5 ).

**Fig. 4:**
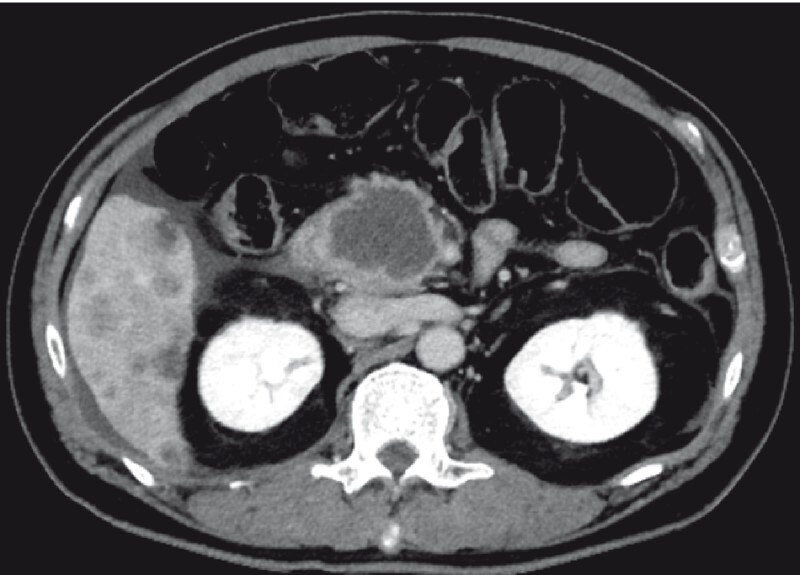
Case 3: Contrast-enhanced computed tomography image indicated a hypovascular pancreatic head tumor, 50 mm in diameter, with multiple liver metastases. It was suspected that the inside of the tumor was necrotic.

**Fig. 5:**
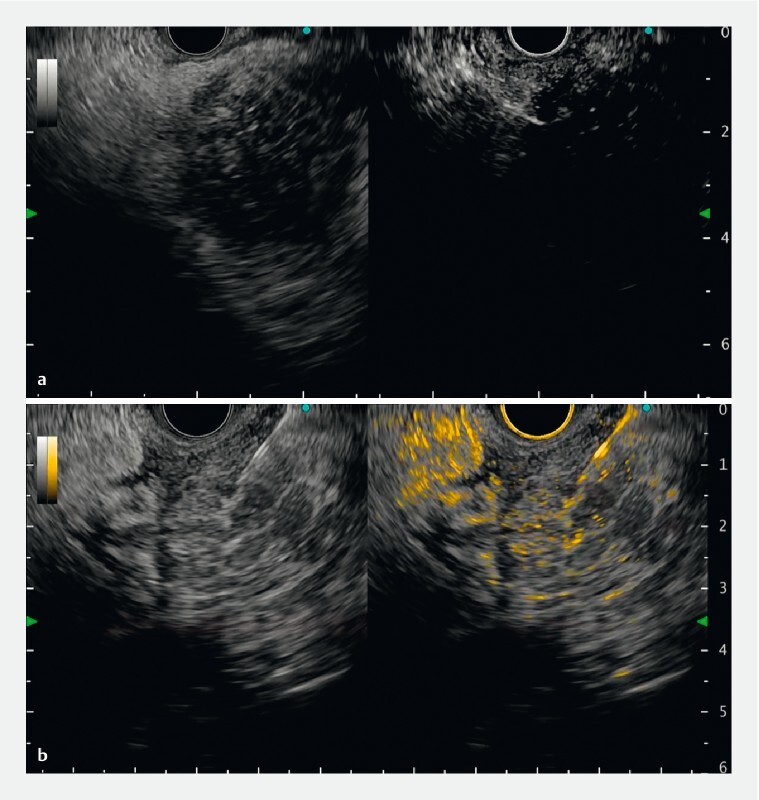
Case 3: **a** There was a large mass lesion in the pancreatic head, which was hypovascular, and no viable location could be recognized by conventional contrast-enhanced endoscopic ultrasound (EUS). **b** In the color overlay mode, the contrast color map was overlaid onto the B-mode, so the lesion and blood flow could be well recognized even after switching to single view. EUS-guided tissue acquisition from the viable area was performed.

Technological advances in endoscopic equipment allow endoscopists to perform the procedure more accurately. The newly introduced color overlay mode may increase accuracy and reduce endoscopists’ stress during EUS procedures.

Endoscopy_UCTN_Code_TTT_1AS

## Notes

Endoscopy E-Videos

https://eref.thieme.de/e-videos
*E-Videos* is an open access online section of the journal *Endoscopy* , reporting on interesting cases and new techniques in gastroenterological endoscopy. All papers include a high-quality video and are published with a Creative Commons CC-BY license. Endoscopy E-Videos qualify for HINARI discounts and waivers and eligibility is automatically checked during the submission process. We grant 100% waivers to articles whose corresponding authors are based in Group A countries and 50% waivers to those who are based in Group B countries as classified by Research4Life (see: https://www.research4life.org/access/eligibility/ ). This section has its own submission website at https://mc.manuscriptcentral.com/e-videos

## References

[1] Gincul R Palazzo M Pujol B Contrast-harmonic endoscopic ultrasound for the diagnosis of pancreatic adenocarcinoma: a prospective multicenter trial Endoscopy 2014 46 373 379 24532350 10.1055/s-0034-1364969

[2] Kamata K Kitano M Omoto S Contrast-enhanced harmonic endoscopic ultrasonography for differential diagnosis of pancreatic cysts Endoscopy 2016 48 35 41 26605974 10.1055/s-0034-1393564

[3] Yamamoto N Kato H Tomoda T Contrast-enhanced harmonic endoscopic ultrasonography with time-intensity curve analysis for intraductal papillary mucinous neoplasms of the pancreas Endoscopy 2016 48 26 34 26561919 10.1055/s-0034-1393563

[4] Krishna S G Rao B B Ugbarugba E Diagnostic performance of endoscopic ultrasound for detection of pancreatic malignancy following an indeterminate multidetector CT scan: a systemic review and meta-analysis Surg Endosc 2017 31 4558 4567 28378082 10.1007/s00464-017-5516-y

[5] Yamashita Y Shimokawa T Ashida R Comparison of endoscopic ultrasonography with and without contrast enhancement for characterization of pancreatic tumors: a meta-analysis Endosc Int Open 2022 10 E369 E377 35433200 10.1055/a-1782-5033 PMC9010094

